# Optically transparent dense colloidal gels

**DOI:** 10.1039/c7sc00901a

**Published:** 2017-05-26

**Authors:** M. Zupkauskas, Y. Lan, D. Joshi, Z. Ruff, E. Eiser

**Affiliations:** a Optoelectronics Group , Department of Physics , Cavendish Laboratory , University of Cambridge , J J Thomson Avenue , Cambridge CB3 0HE , UK . Email: ee247@cam.ac.uk; b Melville Laboratory for Polymer Synthesis , Department of Chemistry , University of Cambridge , Lensfield Road , Cambridge , CB2 1EW , UK; c Collaborative Innovation Center of Chemical Science and Engineering , Institute of Polymer Chemistry , Nankai University , Tianjin 300071 , China

## Abstract

Traditionally it has been difficult to study the porous structure of dense colloidal gels and (macro) molecular transport through them simply because of the difference in refractive index between the colloid material and the continuous fluid phase surrounding it, rendering the samples opaque even at low colloidal volume fractions. Here, we demonstrate a novel colloidal gel that can be refractive index-matched in aqueous solutions owing to the low refractive index of fluorinated latex (FL)-particles (*n* = 1.37). Synthesizing them from heptafluorobutyl methacrylate using emulsion polymerization, we demonstrate that they can be functionalized with short DNA sequences *via* a dense brush-layer of polystyrene-*b*-poly(ethylene oxide) block-copolymers (PS-PEO). The block-copolymer, holding an azide group at the free PEO end, was grafted to the latex particle utilizing a swelling–deswelling method. Subsequently, DNA was covalently attached to the azide-end of the block copolymer *via* a strain-promoted alkyne–azide click reaction. For comparison, we present a structural study of single gels made of FL-particles only and composite gels made of a percolating FL-colloid gel coated with polystyrene (PS) colloids. Further we demonstrate that the diffusivity of tracer colloids dispersed deep inside a refractive index matched FL-colloidal gel can be measured as function of the local confinement using Dynamic Differential Microscopy (DDM).

## Abstract 2

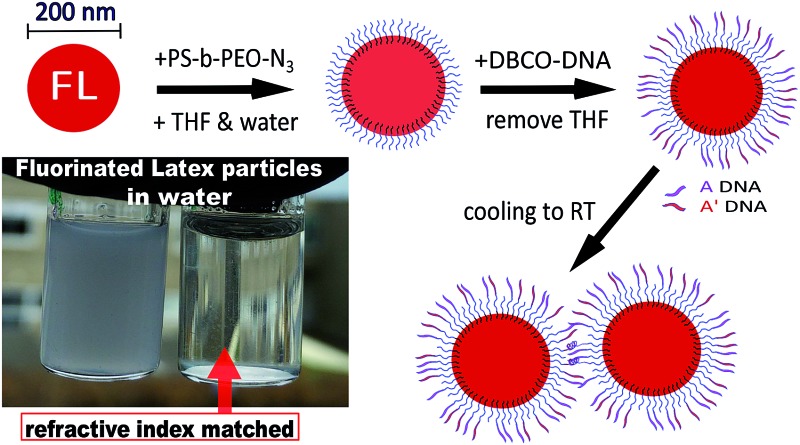
Fluorinated latex particles were synthesized and functionalized with single stranded-DNA, facilitating controlled aggregation into porous gel networks; these can be studied deep into the bulk phase due to refractive-index matching.

## Introduction

Colloidal particles with appropriately designed surface functionalities can readily self-assemble into a range of structures, including crystals, glasses and gels.^1–9^ One possible functionalization in water is the use of DNA as ‘intelligent glue’ due to the selectivity, controllability and thermal reversibility of the bonds between two complementary single-stranded (ss) sticky ends.^4–8,10,11^ Colloidal gels are of great technological importance: they are used in food science, cosmetics, ceramics processing, battery electrode and fundamental soft matter research.^1,12–14^

The majority of experimental research in colloid science is done using polystyrene (PS) and poly(methyl methacrylate) (PMMA) particles. They are relatively easy and cheap to synthesize with a variety of different surface groups rendering them either hydrophobic or hydrophilic. However, already at volume fractions of a few percent, their suspensions become opaque due to multiple-light scattering, making optical probing deep into the bulk phase rather difficult. This is particularly problematic in aqueous solutions, in which the refractive-index difference between water and the polymeric material is large and typically insurmountable. Getting information using small angle X-ray or neutron scattering is possible, so is refractive index matching using organic solvents in dynamic light scattering experiments.^15–17^ There are limitations when working with larger colloids, and only a few colloidal materials can be index matched in non-aqueous solvents. However, some experiments do require optical imaging in aqueous solutions, for example, in the study of microbial transport dynamics through porous media,^18,19^ water purification,^20^ or the solidification process of colloidal latex particles in drying films, which is relevant for processing environmentally friendly paints.^21^

The synthesis of fluorinated latex (FL) particles by Sacanna *et al.* showcased the possibility of index matching polymeric colloidal particles in water.^22,23^ Here we demonstrate that by functionalizing FL particles with DNA *via* block-copolymers using a swelling–deswelling process we are able to make high-volume-fraction gels, which turn completely transparent in sucrose–water solutions, enabling imaging deep inside the structure. In addition, we exploit the specific binding-energy of DNA to construct mixed (FL and PS) core–shell (FL–PS and PSFL) colloidal gels and perform structural studies in order to assess the characteristic pore sizes of the gel as function of height in the partially sedimented gel. Fluorescently labelled PS particles are then used to demonstrate that their diffusivity can be traced deep inside the transparent FL-particle gel and associated to the confinement posed by the varying pore sizes.

## Materials and methods

### Materials

Styrene, potassium persulfate (KPS), sodium dodecyl sulphate (SDS), tetrahydrofuran (THF), dimethyl sulfoxide (DMSO) and dibenzocyclooctyne-sulfo-*N*-hydroxysuccinimidyl ester (DBCO-sulfo-NHS) were purchased from Sigma-Aldrich. 2,2,3,3,4,4,4-Heptafluorobutyl methacrylate (HFBMA) was obtained from Alfa Aesar. The diblock polymer PS-*b*-PEO (*M*_w_ = 6900 g mol^–1^, PS = 1300 g mol^–1^, and PEO = 5600 g mol^–1^) was purchased from Polymer Source, and amine-modified ssDNA from Integrated DNA Technologies. BODIPY® TR-X NHS Ester and BODIPY® 505/515 fluorescent dyes were bought from ThermoFisher Scientific.

### Polystyrene particle synthesis

61.8 mg sodium 4-vinylbenzenesulfonate and 405 mg KPS were added to 300 mL deionized water in a 500 mL round-bottom flask. The mixture was degassed and purged with nitrogen five times. Then it was heated to 70 °C while vigorously stirred with a magnetic stirrer. 15.6 g of styrene was injected immediately afterwards. The reaction was allowed to continue for 24 h and then quenched on ice. The resulting 210 nm diameter polystyrene particles were washed using deionized water by repeated centrifugation.

### Fluorinated latex particle synthesis

The 200 nm diameter FL particles were prepared using emulsion polymerization: 124 mL of deionised water was poured into a 250 mL round-bottom flask. 3.25 mg HFBMA was added together with 125 mg SDS – the mixture was emulsified with a magnetic stirrer under nitrogen atmosphere. The amount of SDS controls the size of the resulting particles. 68 mg KPS was dissolved in 1 mL deionized water and added to the emulsified mixture. While being stirred at 800 rpm, the mixture was heated to 70 °C to initiate the polymerization. After 12 h, the resulting colloidal dispersion was purified by dialysis for 24 h (pore size corresponding to molecular weight *M*_w_ = 30 000 g mol^–1^). The washing procedure was repeated 5 times. The colloid stock was kept refrigerated.

### Functionalization of PS-*b*-PEO with azide groups (PS-*b*-PEO-N_3_)

We followed a protocol described by Oh *et al.* ^24^ for the functionalization of the free PEO end with an azide group, and subsequently dissolved the block copolymer in deionized water and kept it frozen until needed.

### Particle functionalization with PS-*b*-PEO-N_3_ using a swelling–deswelling method

We followed a modified procedure introduced by Oh *et al.* ^24^ 100 μL colloids in deionized water (5% v/v) were mixed with 100 μL PS-*b*-PEO-N3. 200 μL THF was added to PS particles and 100 μL to FL particles (50% and 33% of the total volume, respectively). In addition, 2 μL of BODIPY® dye (1 mg mL^–1^ in DMSO) was added to the mixtures to make the particles fluorescent. The rest of the swelling procedure was unchanged. After the swelling, deswelling and washing, the particles were suspended in deionized water and kept refrigerated.

### Particle coating with DNA using strain-promoted alkyne–azide click reaction (SPAAC)

100 μL 0.5 mM amine-modified DNA in phosphate buffer saline (PBS, pH 7.4, 100 mM NaCl) was mixed with 12 μL 25 mM DBCO-sulfo-NHS (dissolved in DMSO). The mixture was shaken for 12 hours and the DBCO-DNA was purified using Illustra NAP-5 columns (GE). 25 μL 5% v/v 200 nm PEGylated particles were mixed with 25 nanomoles of DBCO-DNA in 1 mL PBS. The mixture was shaken for 24 hours at 60 °C, washed with deionized water and re-suspended in TrisEDTA (TE) buffer. The DNA strands used were: amine-5′- TTT TTT TTT TTT TTT GGT GCT GCG-3′ (called **A**), amine-5′-TTT TTT TTT TTT TTT CGC AGC ACC-3′ (**A′**: sticky end complementary to **A**), amine-5′-TTT TTT TTT TTT TTT ATC TAT CGT A-3′ (**B**), and the complementary amine-5′-TTT TTT TTT TTT TTT TAC GAT AGA T (**B′**). They were not self-complementary, meaning **B**/**B′** sticky ends are not complementary to **A**/**A′**. The melt temperatures were calculated to be *T*_m_(**AA′**) = 37.8 °C and *T*_m_(**BB′**) = 18.5 °C for free strands in solution at 50 mM NaCl.

### Sample loading

The DNA-coated colloids were mixed at desired volume fractions in TE buffer containing 50 mM NaCl and then transferred to a wedge-shaped premade sample chamber which was then sealed with a two-component epoxy glue. Before sample loading, the chambers were cleaned with 3 M NaOH solution thoroughly rinsed with deionized water and then plasma treated to increase their hydrophobicity.^25^

### Imaging

Scanning electron microscopy (SEM) images were taken with a LEO GEMINI 150VP FEG-SEM. Dynamic light scattering (DLS) for particle sizing and Zeta potential measurements were taken with Zetasizer Nano ZS (Malvern). The colloidal gels were imaged in a Nikon Eclipse inverted microscope using a 40 × 0.95 NA dry objective. The temperature was controlled using a home-build Peltier stage. The samples were heated to 65 °C, equilibrated for 10 minutes, and then cooled down stepwise (one step is 1 °C in 30 seconds), while taking epifluorescence snapshots at each step. After the gels were formed, the samples were imaged in a Leica TCS SP5 confocal microscope using a 63× oil immersion objective. *z*-Stacks were taken in both fluorescence channels.

### Image analysis

Chord analysis,^26,27^ a two-point correlation method, was used to obtain separate structural information on the gel and ‘empty’ fluid phase (or pore). For this confocal images taken at a given height *z* in the sample were first processed with a Gaussian filter and binarized following standard procedures employing a home developed Mathematica script.^11^ The resulting binarized image (Fig. 2a) shows regions containing the colloidal gel (white) and regions representing the colloid free aqueous solution (black). Chord distributions were obtained by drawing straight lines with a thickness of one pixel-size through the binarized images both vertically and horizontally. The lengths *r* of the chords passing through either gel or fluid phase were measured and plotted in a histogram of the frequencies *f*(*r*) with which the given lengths appeared (Fig. 2a and b). Samples obtained through dynamic arrest of a sinodally decomposed sample show a histogram with exponential decay *f*(*r*) = *f*(0)exp(–*r*/*λ*), where *λ* is the characteristic decay length of the gel or the pore-sizes respectively.

Differential Dynamic Microscopy (DDM) was done using a Matlab routine developed by S. H. Nathan.^28^ Several 1 minute-long fluorescence movies were taken by focusing at different heights in the sample. In DDM frames, separated by a given time difference, were subtracted, so that only dynamic information of the colloid motion remained. The system's relaxation time, *τ* = (*Dq* ^2^)^–1^, was obtained as a function of the scattering wave vector *q* by Fast-Fourier transforming those difference images and correlating them.^28–31^

## Results and discussion

### Particle synthesis

**Fig. 1:**
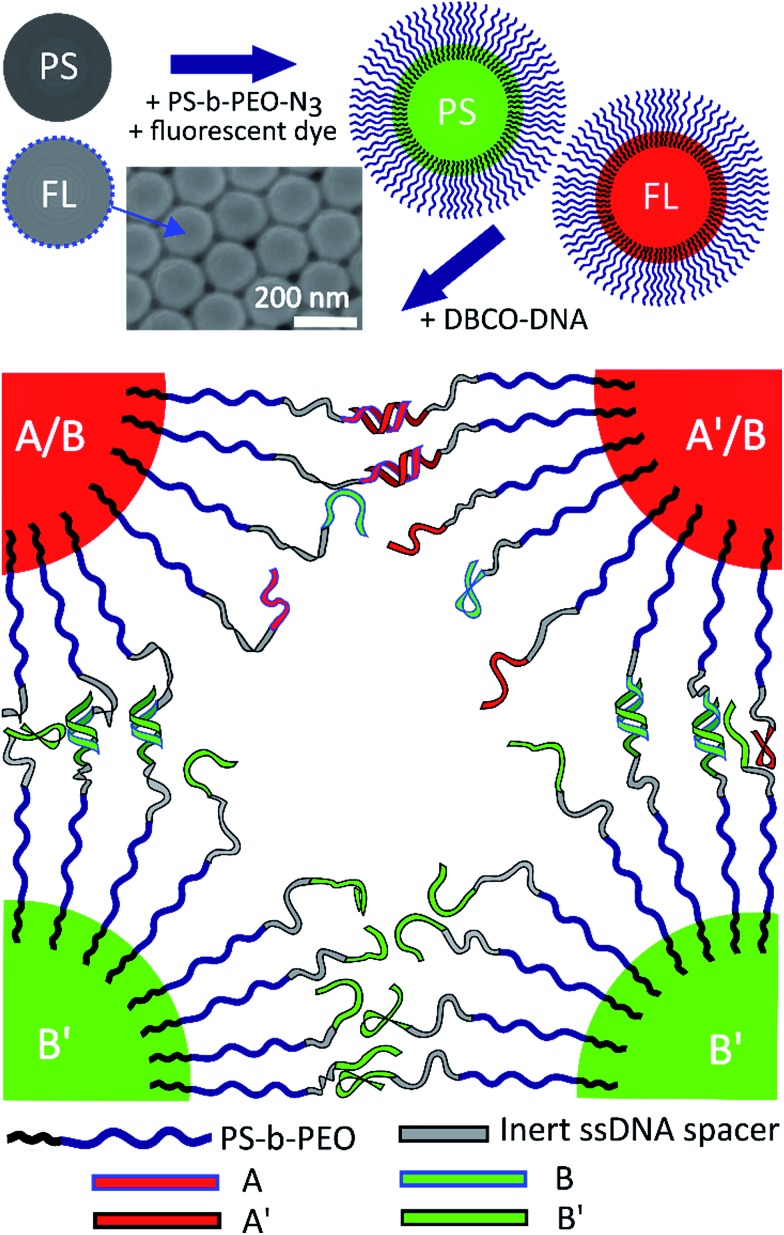
Overview of the coating scheme for PS and FL particles used in the core–shell experiments. Red FL particles form a primary core gel first and green PS particles hybridize to the red gel at a lower temperature.

Fluorinated latex particles with very low polydispersity were synthesized in three different sizes by varying the surfactant concentration, while the HPBMA and KPS concentrations were kept at 0.1 M and 3 mM, respectively. 50 nm, 65 nm and 200 nm PHPBMA particles were obtained using 7 mM, 5 mM and 3.5 mM SDS, respectively. It is important to dialyze rather than centrifuge these colloids, as uncoated FL particles tend to form aggregates if pressed together. As reported by Sacanna *et al.*,^22,23^ these particles have a refractive index of about *n*_FL_ = 1.37 and become optically transparent when dispersed in deionized water containing 24% w/v sucrose, corresponding to *n*_solvent_ ≈ 1.37 at room temperature.^32^ We chose the 200 nm fluorinated spheres for our core–shell gelation experiments, matching their diameter to our PS particles (Fig. 1). The addition of sucrose does not alter the DNA hybridization.^33^

### Single component gels

We first demonstrate the ability to image deep inside a pure FL-colloid gel. DNA functionalized PS and FL samples were prepared and then imaged using confocal microscopy. In both samples, half the particles were coated with **A** DNA, and the other with **A′**. Zeta potential measurements of the bare PS and FL particles revealed an average potential of –90 mV and –46 mV, respectively, changing to about –35 and –22 mV after the surface modification, confirming a dense DNA coating. The FL samples were refractive index matched using sucrose in phosphate buffered saline (PBS), and the particles were labelled with a small amount of red fluorescent dye during swelling. The sealed samples were heated to 70 °C, well above the **AA′** melt temperature, until they reached their equilibrium colloidal-gas phase, and then cooled slowly to room temperature (RT), deep inside the 2-phase region. These slow cooling rates provided us with highly reproducible gel structures generated by arrested spinodal decomposition. The resulting arrested gels showed reliably the same structural characteristics for many melting–cooling cycles.^11^ Confocal *z*-stacks were then taken, again at RT, sampling possible structural changes as a function of sample height *z*.

**Fig. 2:**
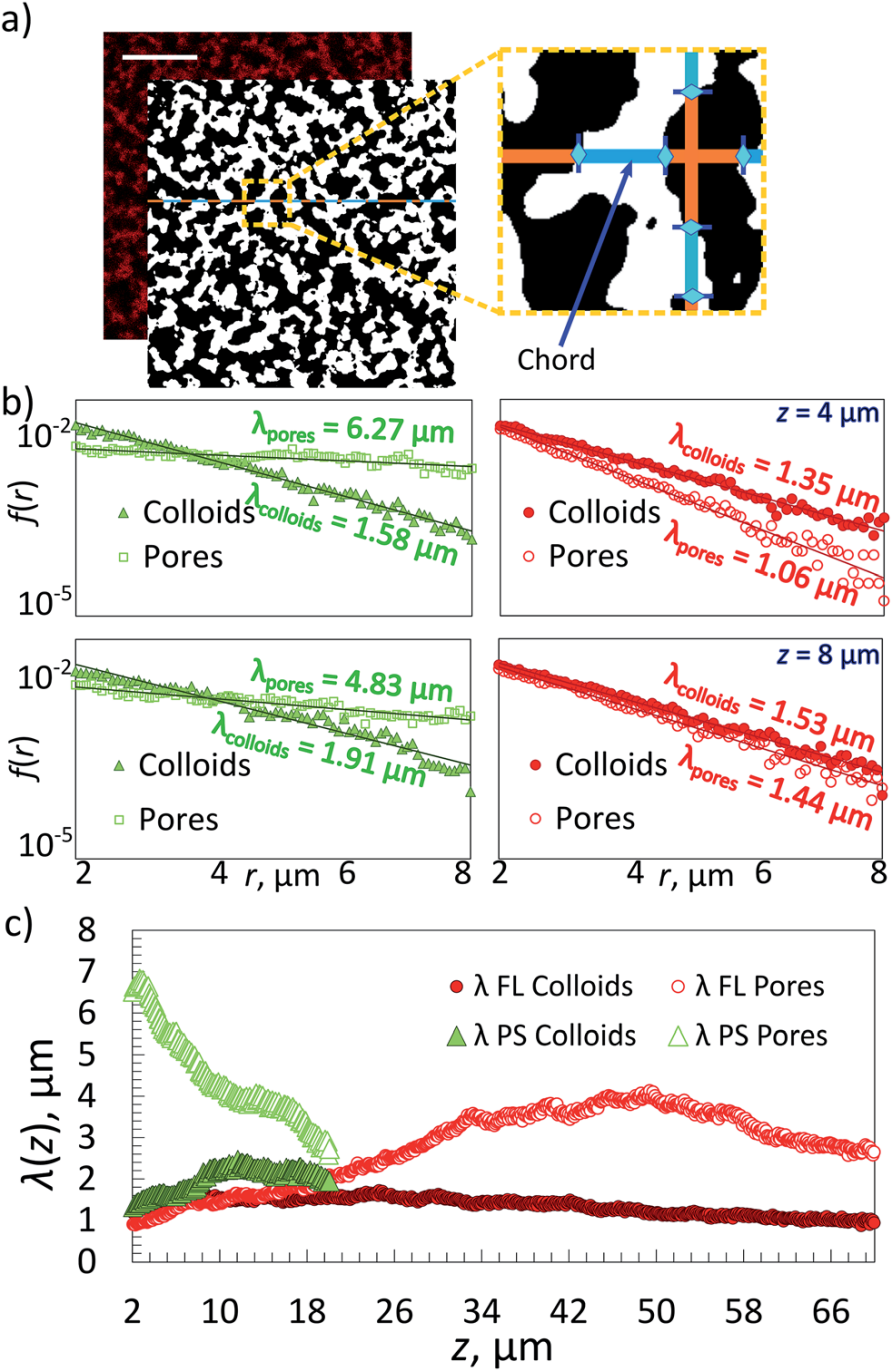
(a) Left: A confocal fluorescence image of a refractive index matched *Φ* ≈ 10% FL gel at *z* = 30 μm and its thresholded and binarized image. Scale bar is 20 μm. Right: Zoom into the thresholded image showing a chord going through the colloid-poor fluid phase (orange) and colloid-rich gel phase (blue). (b) Histograms of chord distributions of a density matched *Φ* ≈ 10% PS-gel (green fluorescent; left) and a FL-gel (red fluorescent; right) at a given height *z* in the sample. The distributions for the respective gels and pores were fitted using an exponential decay with the decay length *λ*. (c) Plot of the characteristic length-scale *λ* as a function of imaging depth *z* of *Φ* ≈ 10% gels, containing either PS or FL particles.

We obtained information on the 3D structure of the gel and the phase-separation mechanism from a method called ‘chord analysis’. In Fig. 2 we illustrate this method for a single-component gel containing a volume fraction of *Φ* ≈ 10% FL colloids. A confocal image taken at height *z* = 8 μm was blurred using a Gaussian filter, thresholded and then binarized and a histogram of the frequencies *f*(*r*) with which chords (lines) pass either through gel or fluid pore-space were plotted. Given that the samples were usually isotropic in the *x*–*y* plane, no preferential direction for the lines were detected. We fitted portions of *f*(*r*) for the gel and the fluid phases separately using an exponential function with a characteristic decay lengths *λ*, which is typical for a kinetically arrested spinodal phase separation.^11,26,34,35^ These length-scales were then plotted against the height *z*, with *z* = 0 being the bottom of the sample chamber (Fig. 2c). Note that because it is almost impossible to align the samples perfectly horizontal in the microscope we only plot *λ*'s for heights starting at about 2 μm and above.

In parallel, we prepared a *Φ* ≈ 10% green fluorescent PS gel with the same DNA, however, in this case we used only 12% w/w sucrose containing buffer solution to density match the PS particles. We followed the same imaging procedure. Note that it was not possible to image the samples deeper than *z* ≈ 14 μm due to multiple light scattering and adsorption stemming from the difference in refractive indices between water and PS (*n*_PS_ ∼ 1.56), while we were able to image the FL samples up to depths of ∼70 μm.

Comparing the two percolating gels we notice that the one made of PS-particles in a density matched medium displays a smaller decay length for the gel-phase than that of the pores at *z* = 8 μm and *Φ* ≈ 10% (Fig. 2b). This is in agreement with previous measurements using 500 nm large PS colloids.^25^ In fact, because of the density matching one would expect to observe the same decay lengths for gels and pores for all heights. However, it appears that the gel-structure becomes ‘less dense’ with increasing height. This can be rationalized by two factors: one is that it remains difficult to perfectly density-match the sample, in particular once the macroscopically large gel has formed – here we overcompensated by making the fluids somewhat denser than the colloids. The second reason is that at this height in such a strongly scattering sample the fluorescence detected in reflection becomes weaker, hence the gel phase becomes darker and increasingly larger errors will be made. In contrast the refractive index matched FL-particle gel can be visualized up to 70 μm deep into the sample. However, because of the density difference between the FL particles and the buffer solution we observe strong sedimentation effects on the gel structure. This is reflected in the equal decay lengths for pores and gel-regions at *z* = 8 μm (Fig. 2b), that suggest a local volume fraction occupied by the colloidal gel is roughly 40%, although the overall value is only 10%. This is also noticed in the decay length of the gel structure that becomes increasingly non-exponential at larger *r* (not shown here). This effect will be discussed further in the following sections.

### FL as the ‘core’ gel

**Fig. 3:**
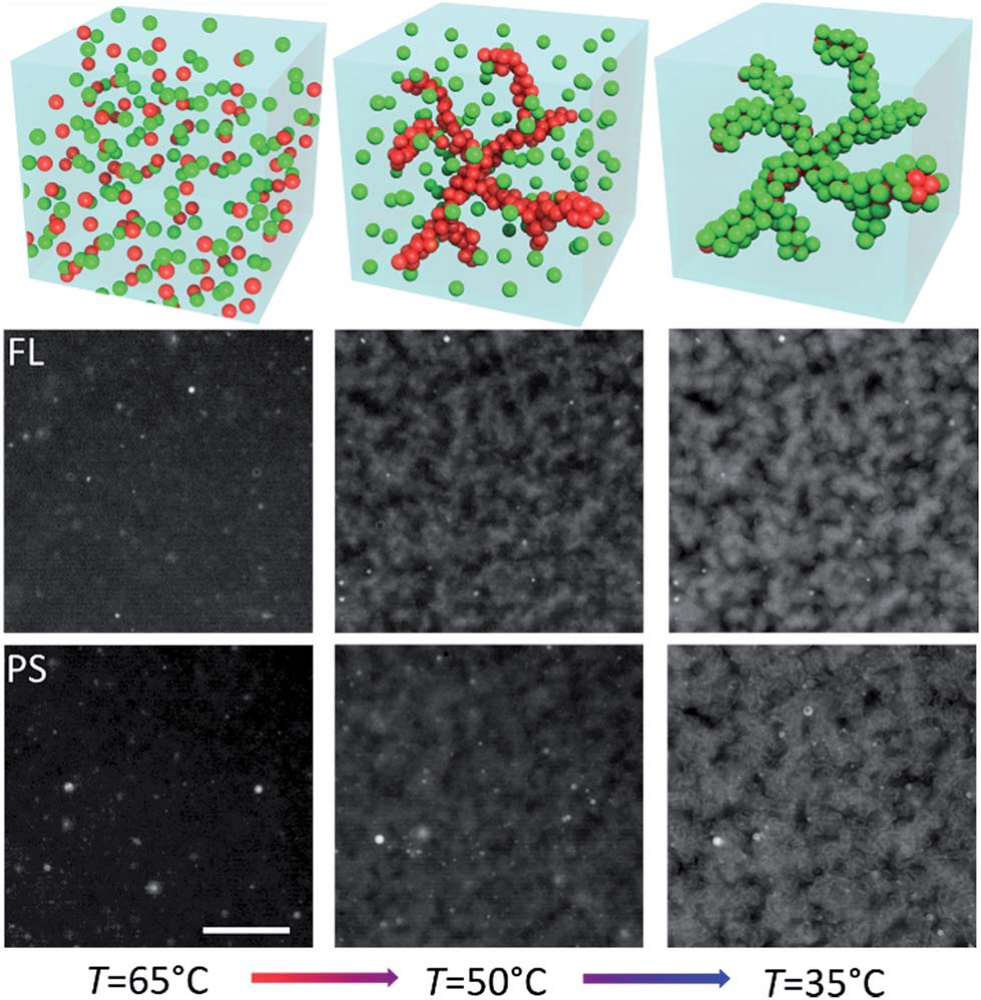
Top: Cartoon showing the binary FL–PS colloid mixture in the gas phase above *T*_m_(FL_core_) (left), slightly below *T*_m_(FL_core_) (middle) and finally below *T*_m_(PS_shell_) (right). Bottom: Epifluorescence snapshot microscope images of FL_core_ (red-labelled, middle) and PS_shell_ (green-labelled, bottom) colloids at different stages of cooling. *Φ*_core_ = *Φ*_shell_ ≈ 5%. Scale bar, 20 μm.

**Fig. 4:**
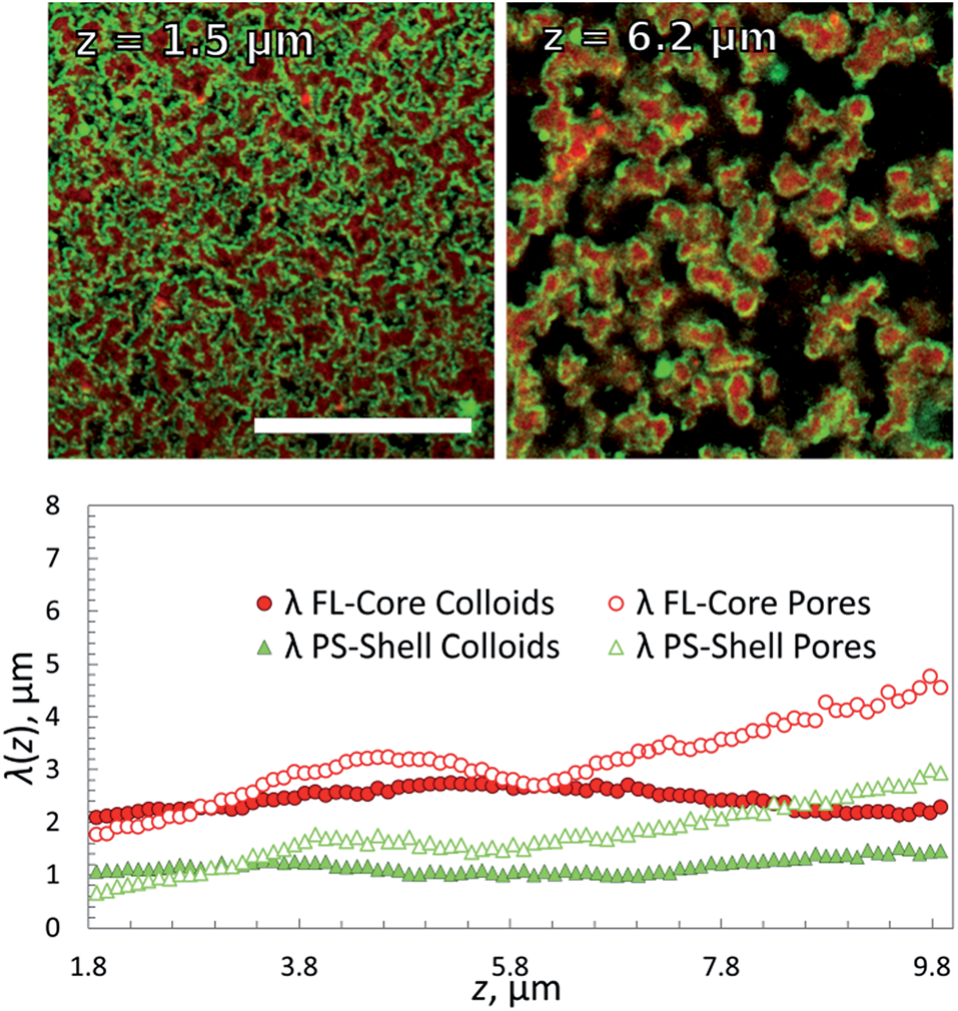
Top: Overlaid confocal fluorescence images for two different heights of a 1 : 1 mixture of primary gel forming FL and coating PS particles with a total volume fraction of 10%. Scale bar, 20 μm. Bottom: Characteristic length-scale, *λ*(*z*), *versus* height *z* in the sample, extracted from chord analysis of the corresponding fluorescent images of the FL (red) and PS (green) system. *Φ*_core_ = *Φ*_shell_ ≈ 5%. Scale bar, 20 μm.

The fluorinated core-gel colloids were made of two batches, one functionalized with **A**/**B** and the other with **A′**/**B**, using equimolar ratios of **A** to **B** and **A′** to **B**, respectively. The intra-species attraction between the fluorinated core colloids was purely due to **A** and **A′** hybridization, while equally sized **B′**-coated PS particles hybridize to the FL-core gel at lower temperatures. For a one-to-one mixture of **A**/**B** and **A′**/**B** coated FL particles we measured a melting temperature *T*_m_(FL_core_) ≈ 58 °C. The **BB′** binding was tested using two batches of PS particles coated with **B′** and **B**, respectively: we found *T*_m_(PS_shell_) ≈ 45 °C. Fig. 3 shows epifluorescence snapshots of a core–shell sample, where the core consists of volume fraction *Φ*_core_ ≈ 5% (2.5% **A**/**B** and 2.5% **A′**/**B**) of FL particles and *Φ*_shell_ ≈ 5% PS (**B′**) particles. Hence, the total colloid-volume fraction was ∼10%, well within the percolation limit.^23^ After heating the sample to *T* = 65 °C, allowing the colloids to mix in the colloidal-gas phase, the sample was cooled below *T*_m_(FL_core_), triggering the hybridization between the red fluorescent FL-particles. A percolating primary gel formed within a temperature range of 1–2 °C, in agreement with previous colloidal gelation experiments.^10,36^ The PS colloids remained in a gaseous phase, visible as green fluorescence occupying the regions depleted of FL particles (Fig. 3, bottom-middle). Further cooling below *T*_m_(PS_shell_) condensation of these green-fluorescent PS particles onto the primary gel was observed (Fig. 4, and 3 in grey scale).

Confocal *z*-stacks were then taken for this system. Characteristic length-scales *λ*(*z*) for the different components in the sample are shown in Fig. 4. *λ*(*z*) for the empty space and the gel were determined for the two fluorescence channels separately. We observe a finer but denser structure for the FL_core_ gel at the bottom of the sample than at the top, as observed in the single FL-gels, which is due to the higher density of the FL particles (*ρ*_HFBMA_ = 1.345 g cm^–3^, *ρ*_styrene_ = 1.05 g cm^–3^): while in the gas phase, both PS and FL particles are homogeneously distributed, both having a similar gravimetric height of tens of microns. When quenched into the 2-phase region, immediately small clusters form due to DNA hybridization (due to spinodal decomposition). Increasingly larger clusters effectively feel a stronger gravitational pull than the individual particles, and start sedimenting. Though, no full compression of the gel structure due to gravity is observed because the DNA bonds between clusters are strong enough to hold much of the open gel network. Evidently the thickness of the coating (solid green dots in Fig. 4) remained 1 μm throughout the sample, as the PS particles do not form clusters on themselves. Note that the PS-coating should be one particle thick,^37^ which would be 200 nm. However, this is of course well below the diffraction limit, nevertheless we see an apparent thickness of 1 μm. This may be due to both the limiting optical resolution and a possible ‘surface-roughness’ of the primary gel. Respectively, the sizes of the ‘empty’ pores were increasing with increasing height *z*.

### PS as the ‘core’ gel

**Fig. 5:**
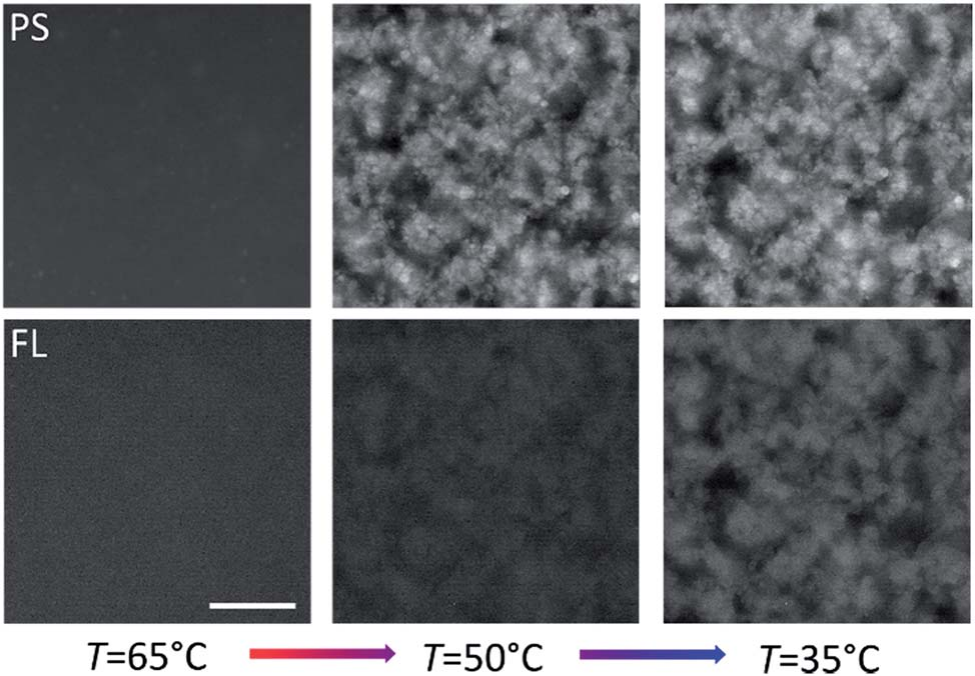
Epifluorescence snapshots of PS_core_ (red-labelled, top) and FL_shell_ (green-labelled, bottom) colloids at different stages of cooling. *Φ*_core_ = *Φ*_shell_ ≈ 5%. Scale bar, 20 μm.

In order to overcome the sedimentation effects we also studied the inverted system by reversing the DNA coating scheme such that the PS colloids formed the primary gel first (*via* **A**/**B** and **A′**/**B** coatings), followed by condensation of the **B′** functionalized FL particles at lower temperatures. Epifluorescence images at different temperatures are shown in Fig. 5. The volume fractions were kept the same as in the previous sample (fraction *Φ*_core_ ≈ 5% (2.5% **A**/**B** and 2.5% **A′**/**B**) PS particles and *Φ*_shell_ ≈ 5% FL (**B′**) particles) but in this case we density matched the PS particles and also the fluorescence labels were swapped.

**Fig. 6:**
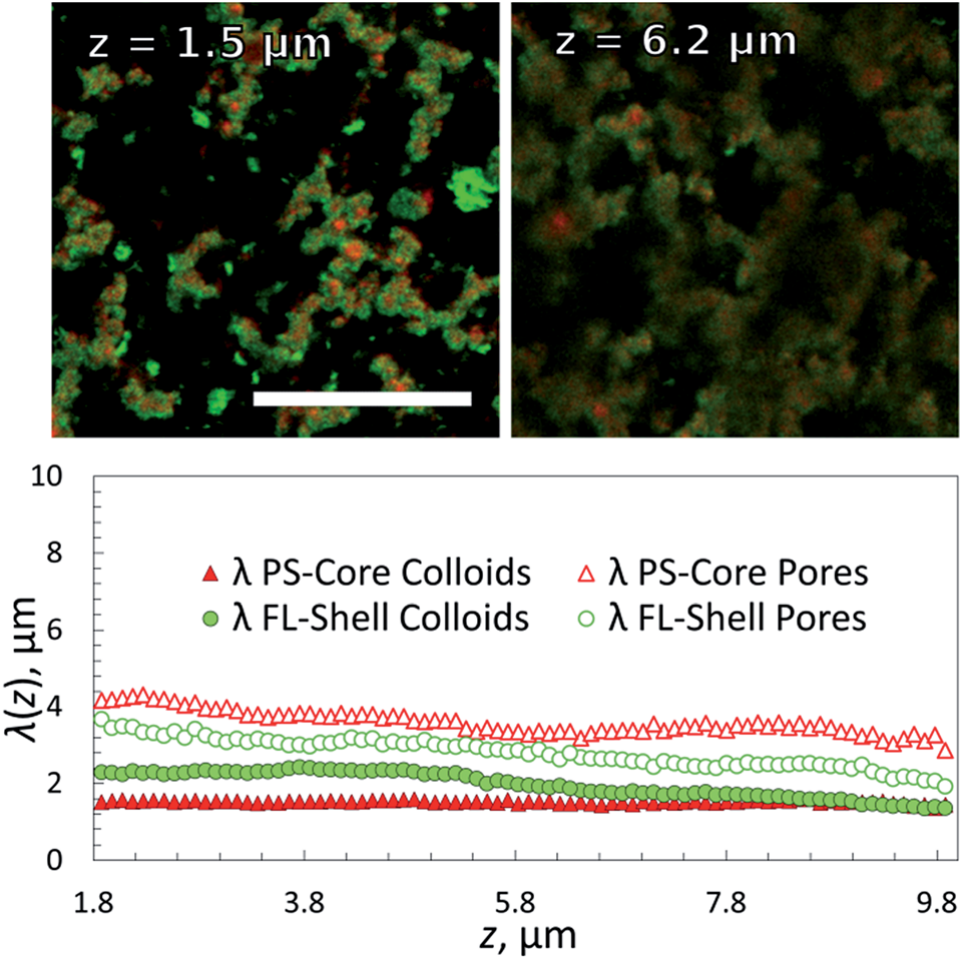
Top: Overlayed confocal fluorescence images for two different heights of a 1 : 1 mixture of PS-forming primary gel coated with FL particles; the total volume fraction was 10%. Scale bar, 20 μm. Bottom: characteristic length-scale, *λ*(*z*), *versus* height *z* in the sample extracted from chord analysis for the PS (red) and FL (green) system. *Φ*_core_ = *Φ*_shell_ ≈ 5%.

Confocal imaging revealed that *λ*_core_ was now constant throughout the height of the structure (Fig. 6) as expected. However, the coating thickness appeared to be larger – the green shell particles were covering most of the red particles in the sliced images, indicating an even higher ‘interfacial roughness’ of the primary structure. Interestingly, we have shown in simulation studies and experiments that a complete coverage of the primary gel by a monolayer of ‘condensed’ colloids requires a 1 : 1 colloid mixture as used in the present study.^37^ This explains the lack of any unbound colloids in solution at room temperature. But the difference in the apparent thickness of the coating remains to be studied in further detail in future. This result demonstrates that we can form 3D gel networks with homogenous pore structure even though we use two very different types of colloidal materials. In particular using the ‘lighter’ PS colloids as scaffolding material we can still distribute the more ‘heavy’ fluorinated particles evenly throughout the system, circumventing strong variations in the gel structure due to gravity.

### Diffusion in a gel network

**Fig. 7:**
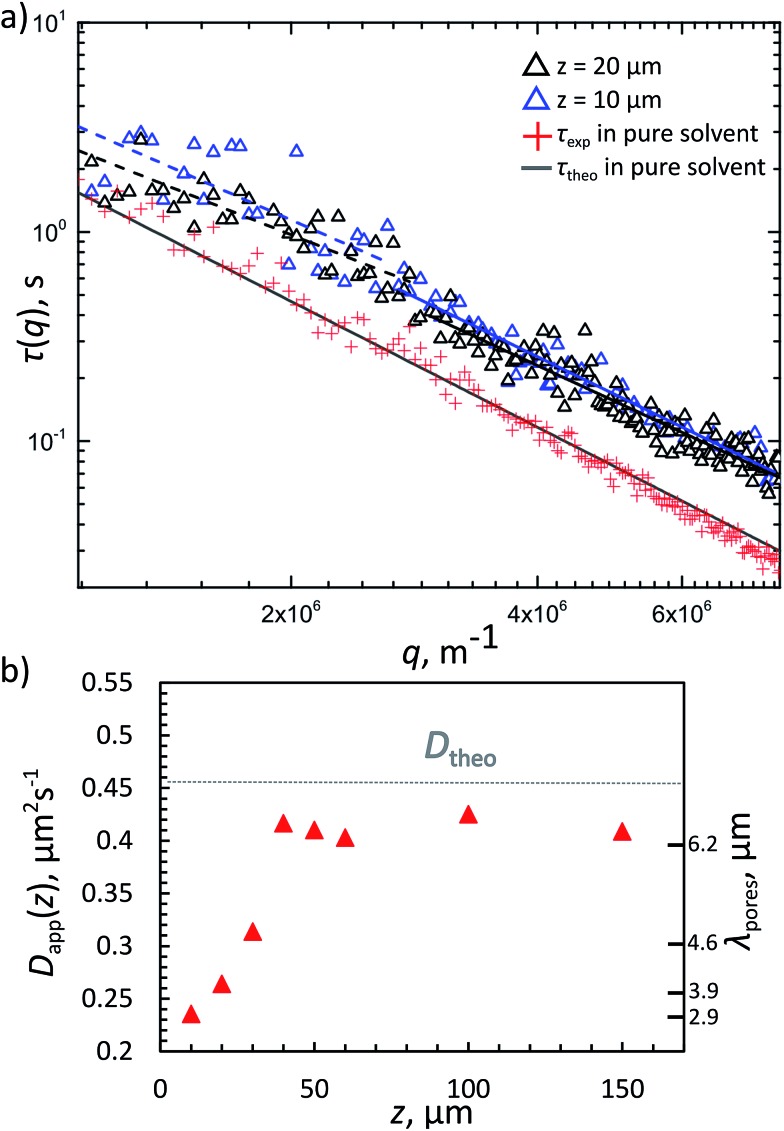
(a) Decay time *τ*(*q*) as a function of the scattering vector *q* extracted from DDM analysis for 420 nm PS tracers in a 10% FL gel in 22% sucrose solution at different heights *z* and their corresponding fits (solid and dashed lines). The theoretical value is given by the relation *τ* = (*Dq* ^2^) ^–1^ (purple dots). (b) Apparent diffusion coefficient *D*_app_(*z*) of the tracers *versus* the height *z* in the sample. The measured *D*_app_(*z*) are also plotted with the corresponding FL gel pore-sizes *λ*_pores_ at different heights.

We also tested the diffusivity of free PS particles in the FL gel at different heights above the sample-container surface, hence for different characteristic pore-sizes *λ*. The 420 nm diameter PS particles were coated with a non-complementary DNA brush with a 69 base-pair long double-stranded DNA as a spacer using the same swelling–deswelling method. The rod-like double stranded DNA has a persistence length of about 50 nm corresponding to 150 base pairs. Hence the steric layer is roughly 20 nm thick, giving the tracer particles an effective diameter of about 440 nm. The coating ensured the particles do not stick to the FL gel. These particles were used as tracers for diffusivity measurements with DDM. The FL gel was prepared as before, with one heating–cooling cycle done before taking videos at room temperature. All samples contained *Φ*_tracer_ = 0.01% tracer beads and 50 mM NaCl in TE buffer with 22 wt% sucrose assuring almost perfect index matching. The fluorescently-labelled gel was characterised as before using confocal microscopy and chord analysis. Many one-minute videos were taken in fluorescent mode of the tracer beads in all samples at different heights starting from the bottom of the sample chamber and analysed in Matlab. From the decay time *τ*(*q*) *versus* the scattering vector *q* plots (Fig. 7a) we extracted the diffusivities of the tracer particles using the relation *τ* = (*Dq* ^2^)^–1^, where *D* is the diffusion constant.

The theoretical result for the 440 nm large particles free in solution (*D*_free,theo_ = 4.5 × 10^–13^ m^2^ s^–1^, considering the viscosity increase due to the dissolved sucrose) were close to the measured values (Fig. 7a). However, when dispersed in a 10% v/v FL gel, DDM measurements taken 10 μm above the lower sample surface show a clear reduction in the apparent diffusivity (*D*_app_ ≈ 2.2 × 10^–13^ m^2^ s^–1^) and a slight deviation from the *q* ^–2^ behaviour. In Fig. 7a the relaxation times *τ*(*q*) of freely diffusing tracer particles (no gel) were fitted with a *q* ^–2^ behaviour, while DDM measurements for 10 and 20 μm above the lower support surfaces could only be fitted with a slightly lower exponent (typically 1.6–1.8). This is in agreement with both theoretical predictions^38^ and recent experimental studied by Cervantes-Martínez *et al.*,^39^ in which the diffusion of a single particle confined inside a water droplet (suspended in oil) was measured as function of droplet size. There too deviations from bulk diffusion due to long-ranged hydrodynamic interactions with the confining walls were observed. These hydrodynamic interactions become increasingly apparent as the confinement – in our case the average pore sizes – becomes smaller. In Fig. 7b we plot the measured apparent diffusion coefficient for different heights *z* away from the bottom surface, together with the corresponding characteristic pore sizes *λ* measured in the same sample. At *z* = 40 μm, the apparent diffusion coefficient reaches values similar to that of the freely diffusing tracer particle. At this height *λ* ≈ 6.2 μm, hence the average pore sizes are about 15 times the diameter of the tracer particles. This is in good agreement with theoretical predictions and previously reported observations for diffusion in confinement (see ref. Cervantes-Martínez^39^ and the references therein). Finally, it is interesting that *D*_app_ reaches almost the free diffusion coefficient but not completely supporting that the FL-network appears evenly dense between *z* = 40–150 μm, and that even at this mild confinements the tracer particles do feel the confining environment.

## Conclusions

We have demonstrated the ability to create optically transparent dense colloidal gels by functionalizing fluorinated latex (FL) particles with DNA using a swelling–deswelling method. Using sucrose to refractive index-match our fluorinated colloids in aqueous media enabled us to image such gels made of 200 nm diameter particles at 10% volume fraction up to ∼70 μm deep into the sample, compared to only ∼15 μm for similarly sized polystyrene (PS) particle gels. In order to overcome the fact that the FL particles have much higher density than the solution leading to sedimentation affecting the resulting gel structure we employed the lighter PS colloids as a primary gel onto which we could condense the FL particles. Thus we were able to form spatially homogenous gels. Finally, we showed the ability to track PS-tracer beads inside an index-matched FL gel, obtaining diffusivity data using Differential Dynamic Microscopy.

## References

[cit1] Manley S. (2005). Phys. Rev. Lett..

[cit2] Lu P. J. (2008). Nature.

[cit3] Cipelletti L. (2003). Faraday Discuss..

[cit4] Mirkin C. a, Letsinger R. L., Mucic R. C., Storhoff J. J. (1996). Nature.

[cit5] Alivisatos A. P. (1996). Nature.

[cit6] Park S. Y. (2008). Nature.

[cit7] Nykypanchuk D., Maye M. M., van der Lelie D., Gang O. (2008). Nature.

[cit8] Zaccarelli E. (2007). J. Phys.: Condens. Matter.

[cit9] Wang Y. (2015). Nat. Commun..

[cit10] Di Michele L., Eiser E. (2013). Phys. Chem. Chem. Phys..

[cit11] Ruff Z. (2015). Faraday Discuss..

[cit12] Mezzenga R., Schurtenberger P., Burbidge A., Michel M. (2005). Nat. Mater..

[cit13] Foffi G., De Michele C., Sciortino F., Tartaglia P. (2005). J. Chem. Phys..

[cit14] Schenker I., Filser F. T., Aste T., Gauckler L. J. (2008). J. Eur. Ceram. Soc..

[cit15] Sirota E. B. (1989). Phys. Rev. Lett..

[cit16] Ashdown S. (1990). J. Am. Chem. Soc..

[cit17] Pusey P. N., van Megen W. (1986). Nature.

[cit18] McDowell-Boyer L. M., Hunt J. R., Sitar N. (1996). Water Resour. Res..

[cit19] Leis A. P., Schlicher S., Franke H., Strathmann M. (2005). Appl. Environ. Microbiol..

[cit20] Yang R.-X., Wang T.-T., Deng W.-Q. (2015). Sci. Rep..

[cit21] van der Kooij H. M., Sprakel J. (2015). Soft Matter.

[cit22] Koenderink G. H., Sacanna S., Pathmamanoharan C., Raşa M., Philipse A. P. (2001). Langmuir.

[cit23] Sacanna S., Koenderink G. H., Philipse A. P. (2004). Langmuir.

[cit24] Oh J. S., Wang Y., Pine D. J., Yi G. R. (2015). Chem. Mater..

[cit25] Varrato F. (2012). Proc. Natl. Acad. Sci. U. S. A..

[cit26] Levitz P., Tchoubar D. (1992). J. Phys. I.

[cit27] Di Michele L. (2013). Nat. Commun..

[cit28] Nathan S. H., DNA directed self-assembly of colloidal systems, University of Cambridge, 2015.

[cit29] Giavazzi F., Brogioli D., Trappe V., Bellini T., Cerbino R. (2009). Phys. Rev. E: Stat., Nonlinear, Soft Matter Phys..

[cit30] Cerbino R., Trappe V. (2008). Phys. Rev. Lett..

[cit31] Bruce D. W., O'Hare D. and Walton R. I., Multi length-scale characterisation, John Wiley & Sons, Ltd., 2013.

[cit32] Charles D. F. (1965). Anal. Chem..

[cit33] Geerts N., Eiser E. (2010). Soft Matter.

[cit34] Levitz P. (1998). Adv. Colloid Interface Sci..

[cit35] Testard V., Berthier L., Kob W. (2011). Phys. Rev. Lett..

[cit36] Biancaniello P. L., Kim A. J., Crocker J. C. (2005). Phys. Rev. Lett..

[cit37] Di Michele L. (2013). Nat. Commun..

[cit38] Happel J. and Brenner H., Low Reynolds Number Hydrodynamics, Springer, Netherlands, 1981.

[cit39] Cervantes-Martínez A. E., Ramírez-Saito A., Armenta-Calderón R., Ojeda-López M. A., Arauz-Lara J. L. (2011). Phys. Rev. E: Stat., Nonlinear, Soft Matter Phys..

